# Tele-Audiology: Current State and Future Directions

**DOI:** 10.3389/fdgth.2021.788103

**Published:** 2022-01-10

**Authors:** Kristen L. D'Onofrio, Fan-Gang Zeng

**Affiliations:** ^1^Department of Hearing and Speech Sciences, Vanderbilt University Medical Center, Nashville, TN, United States; ^2^Department of Otolaryngology - Head and Neck Surgery, Center for Hearing Research, University of California, Irvine, Irvine, CA, United States

**Keywords:** tele-audiology, telemedicine, telehealth, hearing aid, cochlear implant, hearing loss, smartphone, tablet

## Abstract

The importance of tele-audiology has been heightened by the current COVID-19 pandemic. The present article reviews the current state of tele-audiology practice while presenting its limitations and opportunities. Specifically, this review addresses: (1) barriers to hearing healthcare, (2) tele-audiology services, and (3) tele-audiology key issues, challenges, and future directions. Accumulating evidence suggests that tele-audiology is a viable service delivery model, as remote hearing screening, diagnostic testing, intervention, and rehabilitation can each be completed reliably and effectively. The benefits of tele-audiology include improved access to care, increased follow-up rates, and reduced travel time and costs. Still, significant logistical and technical challenges remain from ensuring a secure and robust internet connection to controlling ambient noise and meeting all state and federal licensure and reimbursement regulations. Future research and development, especially advancements in artificial intelligence, will continue to increase tele-audiology acceptance, expand remote care, and ultimately improve patient satisfaction.

## Introduction

Telemedicine is defined as “the delivery of healthcare services and information via high-tech telecommunications technologies” (1). This delivery model has been used in various forms since the late 1950s as a means of providing remote services to underserved populations who would not otherwise have access to care (2). Telemedicine has been adapted to the field of audiology, known as *tele-audiology*, to provide remote hearing screenings, diagnostic testing, intervention, and/or rehabilitation services (e.g., hearing aid adjustment, cochlear implant programming) (3). Recently the COVID-19 pandemic has caused widescale disruption to healthcare services worldwide and has consequently accelerated the need for a remote hearing healthcare service model (4, 5). The present review examines the history and current state of tele-audiology care, while also discussing its current limitations and potential future directions.

In 2021, the World Health Organization (WHO) estimated that 1 in 5 or about 1.5 billion people have some degree of hearing loss. Of those, approximately one-third need hearing healthcare intervention, and 80% live in low- and middle-income nations. Despite the global prevalence of hearing loss, only a fraction of those who could benefit from hearing healthcare services actually receive care (6).

Of hearing aid candidates, Grundfast and Liu (7) reported that the rate of hearing aid adoption is 33%. More recent literature suggests that number is closer to 17% and may be significantly less in low- and middle-income areas, such as Africa at ~10% (8). With respect to cochlear implantation, market penetration in developed countries has been estimated at only 20% (9)—and even lower by more recent data [4.4% (10); <10% (11)]. In developing countries, that number is <1% (9). Indeed, it is estimated that under 15% of cochlear implant (CI) candidates have been implanted worldwide (12, 13).

Failure to receive appropriate intervention comes at an enormous price. The global economic impact of unaddressed hearing loss is $980 billion, annually (6). The impact of inadequate hearing care can also be devasting to an individual's well-being and development. If children experience delays in hearing loss detection and intervention, they may suffer deleterious effects on language development, literacy, social and mental well-being, and overall academic progress (14, 15). Similarly, adults with untreated hearing loss may experience social isolation, reduced quality of life, and poorer academic and/or job performance. To that end, hearing loss has contributed to higher rates of both unemployment and under-employment (16). Moreover, hearing loss is the number one modifiable factor that can reduce or even prevent Alzheimer's disease and dementia (17).

Thus, it begs the question—what most contributes to the lack of care, and how can this be improved? While reasons vary from community-to-community and from individual-to-individual, common factors include cost of services (18–20), a shortage of clinical providers/specialized services (21–26), inconvenience of travel (24, 27), communication barriers (28, 29), and racial/ethnic disparities (30–35). The COVID-19 pandemic, with its associated lockdowns and social distancing requirements, led to mass disruption in the delivery of healthcare services worldwide. For example, Alqudah et al. (36) found that daily hearing aid usage dropped dramatically during the pandemic—a finding potentially caused by limited access to device programming, maintenance and/or repair services, a shortage of hearing aid batteries, and/or the cessation of speech and language therapy sessions. Thus, providers were forced to accelerate implementation of tele-audiology as a means of continuing care during the pandemic. While traditional, in-person care remains the “gold standard,” tele-audiology may mitigate several of the aforementioned challenges, expand the reach of audiological care to underserved populations, and help narrow the gap between those who could benefit from services and those who ultimately receive care.

## Methods

A review of the literature was completed between September 2020—November 2021. The search was primarily conducted by the first author (KD) with additional contribution from the second author (FGZ) using two electronic databases, PubMed and Google Scholar. Eligible peer-reviewed articles were searched for using combinations of keywords including telemedicine, tele-audiology, telehealth, audiology, hearing, hearing aid, cochlear implant, otoscopy, audiometry, DPOAE, ABR, smartphone, tablet. For inclusion, articles were required to address the following: (1) barriers to hearing healthcare, (2) tele-audiology services, and/or (3) tele-audiology key issues, challenges, and future directions. Articles were excluded if they were not peer-reviewed, if they were not written in English, and if they were not directly germane to the topics and purpose of this review. Based on our specified criteria, our literature review identified 70 total articles, including 19 articles on barriers to hearing healthcare, 45 articles on tele-audiology services (4 home-based otoscopy studies, 12 tele-audiology hearing screening studies, including 8 audiometric screening and 4 distortion product otoacoustic emissions (DPOAE) testing studies, 16 tele-audiology diagnostic studies, including 11 audiometry studies and 5 ABR studies, and 13 tele-audiology intervention and rehabilitation studies, including 4 on hearing aids and 9 on cochlear implants), and 6 articles on challenges and future directions.

## Barriers to Hearing Healthcare

### Cost

Hearing technology, including its associated components (e.g., batteries), can be financially burdensome to patients (19). It is recommended that the cost of hearing technology not exceed 3% of the gross national income (GNI) per capita (37). Therefore, with a GNI per capita in the United States of $65,910.00 in 2019, the cost of hearing technology should not exceed $1977.30. With a GNI per capita in Ethiopia of $890.00 in 2020, the cost of hearing technology should not exceed $26.70 (38). However, the cost of CI technology in developing countries can be as much as 30 times one's annual income (20). Thus, despite an exponential increase in CI market size over the past 2–3 decades, this growth has not resulted in a commensurate reduction in unit price. The resulting high unit prices severely limit accessibility and adoption rates of hearing technology (20).

In addition to being a financial hardship for patients, hearing healthcare providers can also be burdened by the high purchase price of testing/programming equipment (e.g., audiometer, hearing aid fitting software, cochlear implant programming software), as well as routine equipment maintenance. In areas that lack repair and/or calibration services, necessary maintenance can be cost prohibitive as it may require sending equipment overseas (18). Thus, cost may not only prevent some patients from seeking out hearing healthcare and/or hearing technology, it may also prevent providers from offering specialized services.

### Providers/Specialized Services

The number of clinical professionals and/or specialized services is disproportionately low in developing countries. Compared with one audiologist per ~25,000 individuals in the United States (39), the proportion of audiologists in developing countries ranges from ~1 per 500,000 to 1 per 6,250,000 individuals (25).

The shortage of providers and/or specialized services also exists in many rural and remote communities in developed countries. Powell et al. (26) cited a lack of audiology providers as a barrier for adults with hearing loss in rural parts of Kentucky, USA. Similarly, Barr et al. (21) reported that children with hearing loss in rural parts of Canada and the United States may experience barriers to specialized services (e.g., hearing aids, cochlear implants, therapy services) when compared to those in urban communities. Children in rural parts of Kentucky were less likely to access specialized healthcare services and were more likely to have a delayed diagnosis compared to those in urban areas (22, 23). With a delay in diagnosis, a delay in intervention is inevitable. To that end, Bush et al. (24) reported that children in rural communities were fitted with hearing aids at a median age of 11 months and cochlear implants at 42 months, while children in urban communities were fitted with hearing aids at a median age of 6 months and cochlear implants at 23 months. Thus, children in rural areas experienced about a 5-month delay for hearing aids, and a nearly 20-month delay for cochlear implantation.

### Travel

Inconvenience, time, and cost associated with travel to an on-site clinic can be a significant barrier to care, especially for those in developing countries or rural areas of developed countries. Bush et al. (24) reported average travel distance for patients in rural communities was 96 miles, compared to just 13.5 miles in urban communities. Further, they reported that the distance a patient lived from hearing healthcare services was moderately correlated (*r* = 0.5) with the delay in both hearing aid and cochlear implant intervention.

While travel distance is often shorter for patients in non-rural areas, unique travel challenges also exist for patients in urban communities. For example, patients in urban communities cite overall health issues, mobility challenges, and/or difficulty accessing transportation as barriers to care—all of which can contribute to a lack of or a reduction in traditional, in-person healthcare visits [e.g., (27)].

### Communication

Patients or families of patients with hearing loss routinely report a lack of communication or information as a barrier to care, especially regarding available services and financial support. In rural Kentucky, primary care physicians were found to have limited resources and knowledge about hearing loss (28), which can be a barrier to appropriate, timely referrals and intervention (29).

### Racial/Ethnic Disparities

Minority groups face additional barriers to care, and importantly, disparities may be present even when minorities have reasonable access to care, have advanced levels of education, and are of high socioeconomic status (31, 40). With respect to hearing healthcare specifically, previous studies have demonstrated significantly higher rates of hearing aid use among White older adults as compared to minority groups (28.6–35.4% of White older adults vs. 10–17.1% of minorities) (30, 32, 34). In pediatric patients, Zhang et al. (35) found a relationship between race/ethnicity and the time delay between a failed newborn hearing screening and initial auditory brainstem response (ABR) testing. The mean interval for White patients was 6.3 months [standard deviation (SD): 5.6 months] compared to 12.3 months (SD: 11.8 months) for racial/ethnic minority patients. The mean difference between White and minority patients was 6.0 months (95% confidence interval: 2.3–9.7 months). Mahendran et al. (33) likewise reported racial disparities in hearing healthcare, specifically that cochlear implant evaluations and implantation rates were disproportionately lower among Black patients. Furthermore, they found that hearing was significantly worse among Black patients at the time of referral for cochlear implantation as compared to their White and Asian counterparts.

## Tele-Audiology Services

### Home-Based Otoscopy

The efficacy of home-based otoscopy is of particular interest, as tele-otoscopy holds significant potential in reducing expenditure and costs associated with travel to a clinic. About 80% of children have at least one episode of acute otitis media (AOM) prior to age 3. In fact, otitis media is the most frequent cause of healthcare visits in the pediatric population. Because AOM diagnosis is heavily dependent upon visualization of the tympanic membrane via otoscopy, home-based otoscopy devices can allow parents/caregivers to complete otoscopy and then transmit videos to a physician for remote diagnosis and treatment recommendations. While the viability of smartphone otoscope use by healthcare professionals has previously been demonstrated [e.g., (41, 42)], its home-based use relies on parents or caregivers to independently perform an otoscopic exam. The ability of a physician to make an accurate diagnosis depends entirely on the quality of tympanic membrane visualization.

Shah et al. (43) evaluated the reliability of the CellScope iPhone device (CellScope, Inc., San Francisco, CA) for at-home use and subsequent remote diagnosis of AOM. Participants included children between the ages of 3 months and 17 years. The results revealed low inter-rater agreement between parent-obtained iPhone video-otoscopy and conventional otoscopy by a physician, and high inter-rater agreement between physician-obtained iPhone video-otoscopy and conventional otoscopy. Thus, tele-otoscopy was able to be completed successfully via use of a smartphone, but only when images/videos were obtained by trained healthcare professionals.

Using the same CellScope iPhone device, Erkkola-Anttinen et al. (44) examined whether parents, following a 60-min training session, could perform home-based otoscopy on children between 6 and 35 months of age. The authors found that, *with instruction*, parents are capable of obtaining adequate video of the tympanic membrane. Importantly, physicians were able to detect or exclude presence of AOM in the majority of smartphone otoscopy videos obtained by trained parents. Further, parents reported that at-home use of smartphone otoscopy was both feasible and easy to perform.

Recently, artificial intelligence algorithms have been developed to improve the sensitivity and specificity of home-based otoscopy, making it a reliable and useful alternative to the current standard care model. Chan et al. (45) utilized a smartphone-based machine learning algorithm to detect middle ear fluid in children between the ages of 18 months and 17 years. The authors report a sensitivity and specificity of 85 and 82%, respectively, which is comparable to that of conventional methods (i.e., tympanometry and pneumatic otoscopy). Importantly, parents demonstrated the ability to use the smartphone-based technology with results comparable to trained clinicians, and similar results were obtained across both Android and iPhone smartphone platforms. These results indicate that a smartphone can be used reliably by both parents and/or trained professionals to detect middle ear fluid. Similarly, Cha et al. (46) used a machine learning model to diagnose a variety of ear diseases from 10,544 images of patient tympanic membranes and external auditory canals. Results offer significant promise, as the authors report accuracy via this method was comparable or even *better than* conventional methods.

### Hearing Screening

#### Audiometric Screening

Lancaster et al. (47) compared conventional hearing screenings with hearing screenings completed remotely using portable audiometers in a group of 3rd graders (*n* = 32). On average, the two test methods produced no significant differences, except for a discrepancy in results for 5 of the 32 children. In such cases, the discrepancies were simply due to the lack of a response at one of the three test frequencies (1, 2, or 4 kHz).

More recently, investigators have examined the reliability of tablet-based, computer-based, and smartphone-based audiometry, but results have been mixed. Khoza-Shangase and Kassner (48) compared test results obtained via conventional audiometry and an iPad method, specifically UHear™, in a group of 86 children. Thresholds were significantly poorer with the iPad method as compared to conventional testing, which the authors attributed to higher ambient noise levels, differences in transducers used, and inadequate calibration. On the other hand, Rourke et al. (49) reported that testing with an iPad audiometer was both reliable and cost effective in 220 children between 5 and 11 years of age. Samelli et al. (50) likewise found results obtained by tablet-based testing to be valid and suggested tablet-based screenings may hold particular promise for use in school settings.

Similarly, Dillon et al. (51) examined use of a computer-based hearing screening program (Sound Scouts) which presents stimuli in the form of a game. Participants included 491 children ages 5–14 years (*n* = 394 with normal hearing, *n* = 97 with known hearing loss), as well as adults with normal hearing (*n* = 50). The screening program tested speech-in-quiet, speech-in-noise, and tones-in-noise. The goal of the study was to investigate whether the program was engaging and held the children's attention, whether it detected hearing loss, and whether it could differentiate between conductive and sensorineural hearing loss types. The authors concluded that sensitivity and specificity was sufficiently high, particularly when all three tests were averaged; however, hearing loss type was only identified correctly in two-thirds of cases. Thus, this program is an appropriate hearing screening tool for children in the 5–14 year old age range, particularly when accompanied by follow-up testing to more accurately determine hearing loss type.

Smartphone-based audiometry applications have also been examined for hearing screening use in recent years. In a study of 6,288 children, Wu et al. (52) examined the validity of a smartphone hearing screening application. They determined that although specificity was high (93%), sensitivity was low (37%), thereby suggesting improvements to sensitivity were necessary before widespread use. However, Swanepoel et al. (53) reported no difference in results between smartphone and conventional hearing screenings so long as the smartphone application could be accurately calibrated with noise monitoring in real-time. To this end, Eksteen et al. (54) demonstrated that hearing screenings using an application on a Smartphone connected to supra-aural Sennheiser HD280 headphones performed by staff with minimal training were affordable and offer a promising large-scale service delivery model.

#### DPOAE Testing

Several studies have examined whether remote synchronous (real-time) DPOAE testing could be completed reliably. Krumm et al. (55) compared DPOAE measures completed in 30 adults via traditional, in-person methods with measures completed remotely (via interactive video and screensharing software). No significant differences were found between the two test methods.

Ciccia et al. (56) likewise examined remote synchronous hearing screenings (pure-tone, DPOAE, and tympanometry) in children 6 years old and younger (*n* = 411) in the United States. Compared to traditional, in-person testing, the reliability was 100% for remote pure-tone and DPOAE screenings, and around 84% for tympanometry. Monica et al. (57) conducted remote synchronous audiometric and DPOAE screenings in school-aged children (*n* = 31) in India using teachers as facilitators and found results similar to traditional, in-person testing. Ameyaw et al. (58) examined remote synchronous DPOAE screenings in a group of newborn infants (*n* = 50) in Ghana. Screenings were completed in-person and remotely by an audiologist via the internet with real-time audio, video, and text messaging between the facilitator and audiologist. Again, no differences were found between the two screening methods. Although these studies provided support for the effectiveness of remote DPOAE screening, they also noted technical challenges, such as high ambient noise levels and slow internet speed in rural areas. Further, they highlighted the need for additional research in difficult-to-test populations (e.g., patients with disabilities).

### Diagnostic Testing

#### Audiometry

Givens and Elangovan (59) compared air and bone conduction thresholds via traditional, in-person audiometry with thresholds completed via tele-audiometry. The audiologist accessed the audiometer at the remote site via an internet connection on his or her own computer. No significant differences were found between traditional and remote testing (mean threshold difference: <1.3 dB for air conduction, <1.2 dB for bone conduction). In more recent years, several additional studies have corroborated these findings, suggesting strong reliability and accuracy between tele-audiometry and traditional audiometry for air conduction (60–62), bone conduction (63, 64), and contralateral masking (60, 61).

While many researchers offer support for the reliable use of tele-audiometry in children (65, 66), others have found that due to increased ambient noise/environmental interference, tele-audiometry resulted in more children failing testing than via conventional audiometry (67). For sites where a conventional audiometer and sound-treated test booth are not available, devices such as the KUDUwave 5000 (GeoAxon, Pretoria, South Africa)—a portable audiometer that “uses insert earphones covered by circumaural earphones fitted with internal and external microphones to monitor ambient noise levels” (68)—have been developed to reduce ambient noise levels.

More recently, smartphone-based automated audiometric testing has been examined, as it presents an affordable, cost-effective testing mechanism. van Tonder et al. (69) compared conventional air-conduction audiometry to air-conduction thresholds determined via hearTest, a smartphone-based application for Android devices. Calibrated supra-aural headphones that monitor noise levels in real-time were utilized, and testing was conducted in a soundproof booth. Of the 95 total participants tested, 94.4% of adult smartphone thresholds were within 10 dB of conventional audiometry thresholds-−98% for adolescents. Using the same smartphone application (hearTest), Sandström et al. (70) sought to examine its accuracy and reliability in low-income communities. Testing was conducted in a non-sound treated environment. Of the 63 total participants tested, 80.1% of adult smartphone thresholds were within 10 dB of conventional audiometry thresholds—threshold agreement was lowest at 500 Hz (69.4%) and highest at 2,000 Hz (88.8%). Sensitivity for hearing loss detection was 90.6%, and specificity was 94.2%. The authors suggest results indicate a satisfactory mean difference between the hearTest smartphone application and conventional audiometry, though additional noise monitoring could improve agreement, particularly in the low frequencies. Thus, the hearTest smartphone application provides a low-cost method for obtaining air-conduction thresholds with sufficient accuracy and reliability; however, thresholds are best-obtained when real-time noise monitoring is incorporated, particularly in settings with unfavorable levels of ambient noise.

Online testing and machine learning will likely further improve not only the efficiency of tele-audiometry but more importantly its diagnostic power. Barbour et al. (71) compared an online machine learning audiogram method with the traditional modified Hughson-Westlake method also completed online. Adults between 19 and 79 years of age (*n* = 21) completed air conduction pure-tone audiometry. Similar threshold estimates were obtained using both methods (mean absolute difference: 3.24 ± 5.15 dB). Thus, online machine learning can be utilized with similar reliability and accuracy with the important benefit of a shorter test duration. Additional advantages include its flexibility for expansion to bone conduction, speech perception, and masking. Crowson et al. (72) utilized deep learning in the form of “Auto Audio,” a proof-of-concept model to interpret diagnostic audiograms. Audiograms consisting of various hearing loss types (e.g., conductive, sensorineural, mixed) were used to train several neural networks. While challenges still remain (i.e., mixed hearing losses were most likely to be misclassified), the authors report that this technology holds promise and may enable an automatic and efficient audiogram interpretation method. Pitathawatchai et al. (73) compared a machine learning algorithm and a common approach (based on slope calculations) for predicting the full audiograms of children with sensorineural hearing loss for cases in which only 1 or 2 thresholds between 500 and 4,000 Hz were labeled. Results indicated that the machine learning approach was not only reliable, but also predicted the full set of thresholds with greater accuracy than the common approach.

#### Auditory Brainstem Response (ABR)

Towers et al. (74) compared ABR results obtained between in-person and remote methods in a group of 15 adults. ABRs were completed using either broadband clicks or tone bursts at 500 and 3,000 Hz. There were no significant differences across test sites; specifically, wave latencies across the two methods were within the clinically acceptable range of variability. Similarly, Ramkumar et al. (75) examined the role of real-time diagnostic tele-ABR in a mobile van with satellite connection, which allowed videoconferencing between an off-site audiologist and a trained on-site facilitator. A total of 30 newborns were tested via tele-ABR, and latency results were comparable to conventional in-person ABR measures.

Dharmar et al. (76) conducted remote diagnostic audiological evaluations in infants who did not pass their newborn hearing screening (*n* = 22) in California and examined parent/caregiver satisfaction, as well as the impact these remote services had on improving what had been a high loss to follow-up rate. The procedure included a case history, video otoscopy, tympanometry, acoustic reflexes, DPOAEs, and diagnostic ABR testing. An audiologist conducted the testing remotely via an on-site facilitator who positioned the otoscope and tympanometry probe, and also prepped the skin and placed the electrodes for ABR testing. Thirteen of the 22 children tested were diagnosed with hearing loss. All parents/caregivers rated the importance of remote audiology services as a 7 on a 7-point Likert scale (7 = “extremely important”). Importantly, *all* infants completed the diagnostic testing with zero loss to follow-up, representing a marked improvement compared to a 22% loss to follow-up rate in the region prior to the study. Thus, a remote option can significantly reduce loss to follow-up rates in infants who fail their newborn hearing screenings and experience barriers to traditional, in-person diagnostic test services. Ramkumar et al. (77) likewise reported that offering a remote option improved the loss to follow-up rates in their examination of remote diagnostic ABR testing among children 5 years of age and under who had previously failed a hearing screening. A third study by Hatton et al. (78) examined remote diagnostic ABR testing in infants who did not pass their newborn hearing screenings (*n* = 102) in British Columbia. Remote testing with an on-site trained facilitator determined that 50 of the 102 children had hearing loss, with efficiency and accuracy being comparable to traditional, in-person testing. Most importantly, remote testing resulted in a significant reduction in travel costs ($91,250). There are clear benefits to offering a remote option, though the current lack of standardized procedures and insufficient information technology support across test centers needs to be addressed prior to widespread usage.

### Intervention and Rehabilitation

#### Hearing Aids

##### Remote Fitting and Verification

Ferrari and Bernardez-Braga (79) compared probe microphone measures completed via traditional, in-person procedures and remotely in a group of 60 adult hearing aid (HA) users. The remote setup consisted of application sharing software, desktop videoconferencing, and an on-site facilitator to place the probe tube. No clinically-significant differences across methods were noted, with differences in real-ear unaided response (REUR), real-ear aided response (REAR), and real-ear insertion gain (REIG) measurements varying by only 0–2.2 dB.

Novak et al. (80) examined HA fittings completed remotely by audiology students and faculty, with nursing students and faculty serving as on-site trained facilitators. The tele-audiology setup included video-conferencing and remote desktop access. As part of this study, 181 patients were fit with hearing aids remotely and had probe-microphone verification completed successfully. Significant improvement in quality of life and communication was reported by the majority of patients.

Pross et al. (81) examined the effectiveness of remote HA services at the Veterans Health Administration. In total, 42,697 veterans were fit with HAs and completed outcome measures, 1,009 of whom did so via tele-audiology and 41,688 via traditional, in-person services. An on-site facilitator was present with the patient, and probe-microphone measures and adjustments were completed by an audiologist via video-conferencing. Hearing aid satisfaction was comparable to traditional, in-person fittings, suggesting that remote fittings may be a viable and cost effective alternative.

##### Self-Fitting Hearing Aids/User Programmable Hearing Aids

The ultimate means of facilitating access to care is “self-fitting” hearing aids, which would allow the patient to control and manage the device independently, without dedicated equipment and without assistance from a hearing specialist. Convery et al. (82) discussed a self-fitting behind-the-ear (BTE) hearing aid with an instant-fit tip (National Acoustics Laboratory version). The device utilizes a tone generator for automated, *in situ* hearing threshold measurements, followed by a prescriptive algorithm for hearing aid programming. However, the success of self-fitting depends not only on accurate threshold measurements [e.g., (83, 84)], but also on accurately identifying medical contraindications (e.g., the presence of a conductive or mixed hearing loss).

An intermediate means is user-programmable hearing aids, which, unlike self-fitting hearing aids, require a previously obtained audiogram, a computer with internet, hearing aid programming software, and/or a hardware interface between the hearing aids and a computer (82). Despite increased risk of inappropriate fittings and a lack of supervision by an audiologist, both self-fitting and user-programmable hearing aids can potentially lower cost, increase accessibility by reducing the need for travel, improve performance and satisfaction by adjusting the hearing aids in the patient's real-world listening environment, and ultimately increase hearing aid adoption rates by giving the patient a sense of personal ownership.

#### Cochlear Implants

##### Remote Programming

Several studies have examined the feasibility of remote cochlear implant programming, specifically seeking to answer the question of whether mapping via tele-audiology is equivalent to traditional, in-person programming.

Ramos et al. (85) compared traditional, in-person and remote cochlear implant programming in five adult CI users. Remote programming was completed using an on-site trained facilitator and internet and/or video conferencing to connect with a remote programmer. Twenty-four different sessions were completed (half in-person and half remote) across four intervals, each separated by 3 months, and both methods produced comparable results with respect to thresholds and word recognition scores. Several follow-up studies have replicated the safety and effectiveness of remote CI programming in both adult and pediatric populations (86–91). These follow-up studies also identified limitations and potential means of improving remote CI programming. For example, Hughes et al. (86) found lower speech recognition scores via tele-audiology, which was likely due to higher ambient noise levels in the absence of a sound-treated test booth. Additionally, when the CI was connected to the programming interface, communication with the patient was difficult since the CI microphone was inactive. Hughes et al. recommend using an alternative method of communication, such as video-conferencing, so speech reading or sign language could be possible.

Schepers et al. (90) found no significant differences between the local and remote fittings in terms of Maximum Comfortable Levels (MCL), Threshold Levels (THR), Impedance Field Telemetry (IFT), audiometry, or speech perception results, except for a slightly longer duration for the remote fitting. Like Schepers et al. (90), Luryi et al. (87) found similar results among 20 adult CI users at the Connecticut Veterans Affairs (VA) Healthcare System. Most importantly, both studies found that patients were highly satisfied with remote programming, which is a reliable and cost effective means of providing follow-up care to patients in remote areas or those with limited mobility.

##### Self-Fitting Cochlear Implants

Meeuws et al. (92) examined the feasibility of an autonomous “self-fitting” method in adult CI users (*n* = 6). Study participants completed a self-fitting session, including audiometry and spectral discrimination testing, 2 weeks after initial traditional, in-person activation. An artificial intelligence software system (FOX) was used for interpretation, analysis, and map recommendation. Specifically, it analyzes the test results and the patient's current map, then calculates predicted outcomes with alternative maps. The alternative map with the best predicted outcome is recommended to the patient. Importantly, this method does not completely preclude the role of an audiologist; a CI audiologist was still required to review the recommended map. Following programming of the new map, participants were then re-tested again after 2 months. A questionnaire was also completed. Four of the six participants were able to complete all tests without any additional assistance from the audiologist. Four were fitted with a new map without physical intervention. All six participants reported feeling comfortable with the autonomous process, but initial audiologist supervision may be required or preferred.

## Key Issues

Recent literature has shown that tele-audiology can be completed accurately and reliably; however, additional measures and regulations *specific to the provision of remote care* are necessary. Thus, prior to providing remote services, audiologists must be familiar with the unique licensure, reimbursement, and privacy/security regulations required for tele-audiology services.

### Licensure

Current policy with respect to tele-audiology stipulates that the site of service is determined by the patient's physical location. Thus, an audiologist must be licensed in both the location where services are provided from *and* the location the patient is in when services are received. At present, American Speech-Language-Hearing Association (ASHA) is exploring licensing options that would facilitate multi-state service delivery (3). No global licensure agreement currently exists, thus limiting the usage of tele-audiology across countries or regions.

### Reimbursement

Tele-audiology payment and coverage is variable across state, federal, and commercial payers (e.g., private health insurance, Medicare, Medicaid). For example, while commercial payers and Medicaid have the discretion to provide coverage for tele-audiology services, Medicare does *not* consider audiologists to be eligible telehealth service providers (3). Thus, it is imperative that audiologists confirm billing and coverage policies prior to providing tele-audiology services.

### Privacy and Security

Tele-audiology services must adhere to state, federal, and international regulations with respect to patient privacy and security, particularly that which includes transmission and storage of patient data. Tele-audiology providers must abide by the same regulations applicable to traditional, in-person services. Current federal legislation includes (1) Health Insurance Portability and Accountability Act of 1996 (HIPAA), (2) Health Information Technology for Economic and Clinical Health Act of 2009 (HITECH), and (3) Family Educational Rights and Privacy Act of 1974 (FERPA) (3). It is possible that state requirements may be more rigorous than those at the federal level; thus, it is important for audiologists to familiarize themselves with both federal and state-specific guidance prior to providing tele-audiology care.

Additional important considerations include security of patient rooms, security of electronic documents, security of telecommunications, identification of all individuals present in the rooms at both locations, and documentation of informed consent from the patient. The informed consent document should describe how tele-audiology services may be different from traditional, in-person care, the equipment to be used, the patient's right to switch to traditional, in-person services at any point (if available), modifications (if any) to clinical protocols and procedures, and any potential issues with patient confidentiality. In order to ensure patient confidentiality, the audiologist must be familiar with state and federal regulations regarding electronic storage of patient data, privacy protections (e.g., firewalls, encryption, VPN), configuring software and hardware for use with firewalls, encryption, or VPN, and policies for breach notification (3).

## Discussion

Taken together, the current body of literature suggests that tele-audiology provides a viable service delivery model. Remote hearing screenings, diagnostic testing, intervention, and rehabilitation can be completed safely and effectively, in both children and adults. Moreover, the accurate provision of tele-audiology care has been demonstrated both in developed and developing countries. Benefits of such services include but are not limited to improvement in loss to follow-up, reductions in travel time and costs, and improved access to services not otherwise available in one's physical location.

### Challenges and Future Directions

Still, tele-audiology is not without its logistical challenges. Several modifications to the traditional testing paradigm may be needed in order to successfully implement remote testing. For example, the presence of ambient noise during testing can reduce the accuracy of results. While use of a soundproof or sound-treated test booth is ideal, this may not be feasible in all circumstances. To improve diagnostic accuracy in the absence of a sound-treated environment, headphones with real-time ambient noise monitoring are recommended, as well as detailed instructions for proper headphone use by the patient (93). Further, different protocols may need to be developed for different age groups and for those with varying degrees of hearing loss. For CI care specifically, offering alternative communication strategies (text messaging, video-conferencing, or sign language) may be particularly useful for this population.

Access to digital technology also presents an ongoing challenge. While tele-audiology could help narrow the healthcare gap evident in many underserved communities, it also runs the risk of exacerbating existing inequalities and amplifying the “digital divide” (94, 95). The current digital divide most negatively impacts racial/ethnic minorities, individuals of low socioeconomic status, those in rural areas, and the elderly. For example, while rural communities are very much in need of telehealth opportunities due to a shortage of physicians that is higher than the national average, fewer than 50% of rural households actually have broadband access (96, 97). Thus, a telehealth option is only practical and realistic for less than half the rural community. Further, in order for tele-audiology care to be reliable, the remote test site's internet connection must be strong enough to support real-time communication and data transfer, particularly if a synchronous service delivery model is utilized. In other words, it's not enough to simply have *access* to digital services, the *quality* of the services must be sufficiently reliable (95). Clearly there are a number of logistical barriers that must be overcome to ensure patients in *all* communities have equitable access to high-quality tele-audiology services.

Digital literacy, on both the part of the provider and the patient, presents another challenge. For example, downloading and setting up a mobile application can be quite daunting for those unfamiliar or uncomfortable with digital technology. During the COVID-19 pandemic, social distancing and other safety provisions forced many patients and healthcare providers to rely on computer programs and other mobile tools that may have been beyond their digital literacy level. In the future, formal assessment of a patient's digital proficiency will allow the provider to meet the patient at their comfort level and tailor online intervention accordingly. Questionnaires validated for assessing digital proficiency include the Mobile Device Proficiency Questionnaire (MDPQ-16) and the Computer Proficiency Questionnaire (CPQ-12). The MDPQ-16 consists of 8 domains (mobile device basics, communication, data and file storage, internet, calendar, entertainment, privacy, and trouble shooting and software management) with 16 questions. The CPQ-12 consists of 6 domains (computer basics, printer, communication, internet, calendar, and entertainment) with 12 questions (98).

Additional challenges include reluctance or even resistance from payers, as well as the increased regulation required, particularly with respect to licensure, reimbursement, and privacy and security. Specifically, remote care must be delivered in accordance with clinical guidelines, payer policies, and state and federal law. Further, audiologists must ensure that tele-audiology clinical care is equivalent in quality to care delivered in-person. While undoubtedly necessary, the additional levels of regulation and requirements may, at least in the short-term, serve as hurdles to the widespread implementation of tele-audiology care.

Future advancements in technology, especially in artificial intelligence, may help facilitate the provision of remote care and may accelerate the adoption of tele-audiology services worldwide at affordable costs (99). To date, artificial intelligence has been implemented in a CI software system (FOX) to allow for autonomous “self-fitting” of the CI device, as well as home-based otoscopy. Future applications of such technology may include commercially-available diagnostic testing (e.g., conventional audiometry) and commercially-available “self-fitting” or even “cognitive-controlled” hearing aids.

## Conclusion

Recent research shows that tele-audiology services can increase patient accessibility and engagement, improve loss to follow-up rates, and reduce cost and travel time. Furthermore, remote services can be completed in a manner that is safe, valid, reliable, and satisfactory. Still, logistical challenges do remain. For example, careful attention must be given to controlling ambient noise (particularly when testing is completed in the absence of a sound-treated test booth), modifications to current testing procedures may be needed to tailor to the provision of remote care, and regulatory and reimbursement hurdles need to be overcome before tele-audiology may be implemented on a widescale. The current COVID-19 pandemic has caused mass disruption to the delivery of healthcare services and has consequently accelerated the pace of development and acceptance of tele-audiology. Future research, including advancements in artificial intelligence, will continue to improve not only the effectiveness and efficiency of tele-audiology services but also most importantly, patient acceptance and satisfaction.

## Author Contributions

KD'O completed a review of the existing tele-audiology literature between September 2020–November 2021, wrote the initial draft of this manuscript, and reviewed and edited the final version. F-GZ provided input on topic coverage, wrote portions of the final manuscript, and reviewed and edited the final version. All authors contributed to the article and approved the submitted version.

## Funding

This study was supported in part by NIH NIDCD (3R01DC015587).

## Conflict of Interest

F-GZ owns stock in Axonics, Dianavi, Nurotron, Syntiant, Velox, Voibook, and Xense. The remaining author declares that the research was conducted in the absence of any commercial or financial relationships that could be construed as a potential conflict of interest.

## Publisher's Note

All claims expressed in this article are solely those of the authors and do not necessarily represent those of their affiliated organizations, or those of the publisher, the editors and the reviewers. Any product that may be evaluated in this article, or claim that may be made by its manufacturer, is not guaranteed or endorsed by the publisher.
